# Accidents That Have Been Observed to Follow Thoracentesis by Aspiration Practiced for the Removal of Pleuritic Effusion

**Published:** 1880-11

**Authors:** 


### ACCIDENTS THAT HAVE BEEN OBSERVED TO FOLLOW THORACENTESIS BY ASPIRATION PRACTICED FOR THE REMOVAL OF PLEURITIC EFFUSION.

N. P. Dundridge, M. D., Cincinnati *Lancet and Clinic*, Jan. 3, 1880, thus closes an article under the above caption. The statement that this operation, mentioned in the caption, is a trivial operation, entirely devoid of danger, is to be most strongly condemned, while the practice which would undertake its performance without due regard to the conditions and surroundings, which would render accessible the most efficient means for combating any unpleasant consequences which might arise, is certainly not justifiable. Syncope has developed half an hour or more after aspiration has been performed, so that the operation should only be undertaken when complete rest and repose can be secured after its performance, for the least exertion might determine an accident, otherwise avoidable, which may prove fatal. The doctor feels himself justified in formulating the following as the accidents which have followed thoracentesis for pleuritic effusions. Some of these may be considered as mere concomitants of the puncture. Others must be held to be more or less dependent upon the operation or the measures by which it was followed: Syncope, fatal or transient, due either to reflex action or to the paralyzing influence on the heart-walls of the sudden removal of the pressure of the effusion; convulsions dependent upon reflex action or due to minute emboli in the cerebral vessels; pulmonary congestion and œdema, suddenly developed, either with or without the rapid accumulation of serous exudation into the bronchial tubes and producing asphyxia; embolic obstruction of the pulmonary artery of the sound lung; embolic processes in various organs and of various grades of severity, which have their origin in clots previously in the pulmonary veins of the compressed lung. These last, when they occur at the time of or soon after the operation, may have been excited by it. When they develop days afterwards, they must be held as incidents of the original trouble, and in no way connected with the operative measures taken. In this *resume* mention of a transformation of a serous into a purulent effusion is purposely omitted, because it was thought that the proof is insufficient to hold the operation responsible for the change from serum to pus. No case of serious accident from wounding the lung seems to be recorded. While the conclusions are not new, the doctor thinks that they will bear repetition, and that surgeons cannot be too careful in undertaking this operation.—*Detroit Lancet.*

